# The post‐translational regulation of transcription factor EB (TFEB) in health and disease

**DOI:** 10.15252/embr.202357574

**Published:** 2023-09-20

**Authors:** Michael Takla, Swati Keshri, David C Rubinsztein

**Affiliations:** ^1^ Department of Medical Genetics, Cambridge Institute for Medical Research (CIMR) University of Cambridge Cambridge UK; ^2^ UK Dementia Research Institute, Cambridge Institute for Medical Research (CIMR) University of Cambridge Cambridge UK

**Keywords:** autophagy, lysosome, mitochondria, post‐translational modification, TFEB, Membranes & Trafficking, Molecular Biology of Disease, Post-translational Modifications & Proteolysis

## Abstract

Transcription factor EB (TFEB) is a basic helix–loop–helix leucine zipper transcription factor that acts as a master regulator of lysosomal biogenesis, lysosomal exocytosis, and macro‐autophagy. TFEB contributes to a wide range of physiological functions, including mitochondrial biogenesis and innate and adaptive immunity. As such, TFEB is an essential component of cellular adaptation to stressors, ranging from nutrient deprivation to pathogenic invasion. The activity of TFEB depends on its subcellular localisation, turnover, and DNA‐binding capacity, all of which are regulated at the post‐translational level. Pathological states are characterised by a specific set of stressors, which elicit post‐translational modifications that promote gain or loss of TFEB function in the affected tissue. In turn, the resulting increase or decrease in survival of the tissue in which TFEB is more or less active, respectively, may either benefit or harm the organism as a whole. In this way, the post‐translational modifications of TFEB account for its otherwise paradoxical protective and deleterious effects on organismal fitness in diseases ranging from neurodegeneration to cancer. In this review, we describe how the intracellular environment characteristic of different diseases alters the post‐translational modification profile of TFEB, enabling cellular adaptation to a particular pathological state.

## Graphical abstract

This review discusses how different diseases, from neurodegeneration to cancer, alter the post‐translational modification profile of TFEB, thus enabling cellular adaptation to particular pathological states.

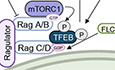

## Introduction

The microphthalmia/transcription factor E (MiT/TFE) family of basic helix–loop–helix leucine zipper (bHLH‐Zip) transcription factors comprises four evolutionarily conserved members: microphthalmia‐associated transcription factor (MITF) and transcription factors EB (TFEB), EC (TFEC), and E3 (TFE3; Rehli *et al*, 1999; Hallsson *et al*, 2004; Steingrímsson *et al*, 2004).

Of all MiT/TFE family members, TFEB has been the most thoroughly implicated in the transcriptional regulation of a diverse range of physiological functions, ranging from lysosomal and mitochondrial biogenesis to macro‐autophagy and host immunity (Sardiello *et al*, 2009; Settembre *et al*, 2011; Samie & Cresswell, 2015). As such, TFEB is a crucial component of cellular adaptation to, and survival in the face of, nutritional, oxidative, lysosomal, mitochondrial, and infectious stressors.

The role of TFEB in disease thus depends on the combination of: (i) selection pressures that arise within the affected tissue (s), which determine the predominant physiological role of TFEB necessary for maintaining cellular fitness; and (ii) the organismal context of the disease, which determines whether changes in the survival of the affected tissue (s) owing to TFEB loss‐ or gain‐of‐function benefit or harm the organism as a whole.

In this review, we describe how post‐translational modifications partly account for the apparently paradoxical protective and deleterious effects of TFEB on organismal fitness in diseases ranging from neurodegeneration to cancer, by acting as molecular barcodes that reflect the selection pressures characteristic of a particular pathological state.

## Evolution, structure, and transcriptional mechanism of TFEB

The MiT/TFE family of transcription factors likely derives from a common ancestor gene that has undergone multiple rounds of duplication events and subsequent functional specialisation (Bouché *et al*, 2016; La Spina *et al*, 2021). Unsurprisingly, therefore, TFE3 and MITF regulate many of the lysosomal and autophagic genes controlled by TFEB (Motyckova *et al*, 2001; Hershey & Fisher, 2004; Meadows *et al*, 2007; Sardiello *et al*, 2009; Settembre *et al*, 2011; Martina *et al*, 2014; Ploper *et al*, 2015; Pastore *et al*, 2017). Notably, TFEC is unique among MiT/TFE family members in repressing, rather than promoting, transcription (Zhao *et al*, 1993). Although the relative importance of each MiT/TFE family member within a particular tissue depends on their abundance therein (Goding & Arnheiter, 2019; Di Malta *et al*, 2023), their gene regulatory networks exhibit redundancy, and neither TFE3 nor MITF have been as extensively studied as TFEB in the transcriptional regulation of as wide a range of physiological roles.

**Figure 1:**
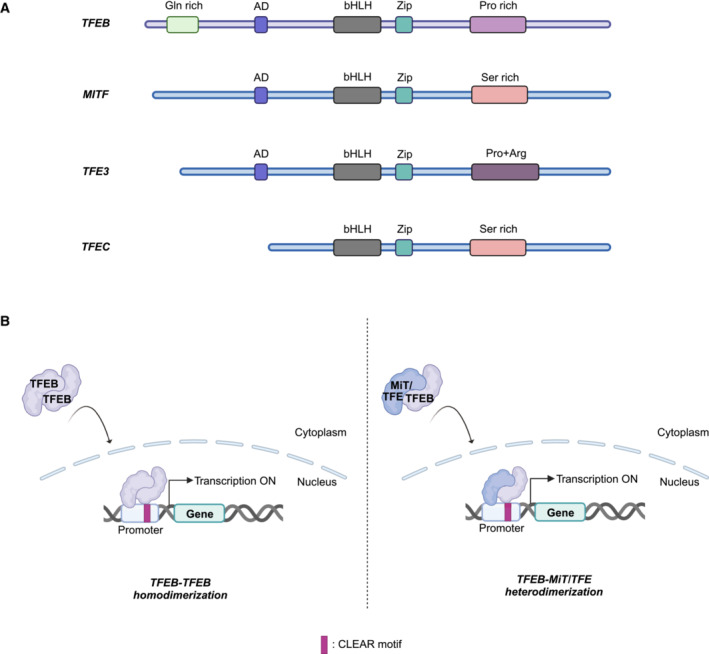
The MiT/TFE family as transcriptional regulators of a network of genes containing CLEAR motifs in their promoters TFEB can homodimerise, or heterodimerise with other MiT/TFE family members, to bind CLEAR elements within the promoters of the genes whose expression it tends to induce. Created with BioRender.com.

While sharing an identical DNA‐binding basic domain and highly similar helix–loop–helix (HLH) and leucine zipper (Zip) regions with other MiT/TFE family members, TFEB is singular in possessing a disordered Gln‐rich N‐terminus (Fig 1A). The HLH and Zip regions permit the dimerisation of TFEB with other HLH‐ and Zip‐containing proteins, while the activation domain (absent in TFEC) enables upstream regulation.

Unusually for a bHLH‐Zip transcription factor, TFEB binds to DNA as either a homodimer or a heterodimer with other MiT/TFE family members, but not with extrafamilial bHLH‐Zip proteins, owing to a conserved kink within the leucine zipper (Hemesath *et al*, 1994; Pogenberg *et al*, 2012). However, like other bHLH‐Zip transcription factors, homo‐ or heterodimeric TFEB has an affinity for an asymmetric E‐box‐like 10 base‐pair (bp) motif (5′‐GTCACGTGAC‐3′), termed the “coordinated lysosomal expression and regulation (CLEAR)” element, found within 200 bp of the transcription initiation site in the promoters of the genes to which TFE3 and MITF are recruited (Aksan & Goding, 1998; Wenger *et al*, 2005; Sardiello *et al*, 2009; Martina *et al*, 2014; Möller *et al*, 2019; Fig 1B). Once bound to CLEAR motifs within the promoter, TFEB undergoes liquid–liquid phase separation (LLPS) to form condensates to which the mediator complex is recruited, facilitating transcription of the gene downstream (Chen *et al*, 2020).

## Regulation of TFEB activity by post‐translational modifications and protein–protein interactions

As for any transcription factor, the activity of TFEB is a function of its: (i) abundance; (ii) localisation to the nucleus; and (iii) capacity to bind the promoters of genes therein.

The activity of TFEB can, therefore, be modulated by transcriptional regulation of its own expression (Sardiello *et al*, 2009; Cesana *et al*, 2023), and a newly identified splice variant of the TFEB‐encoding mRNA, which generates a small TFEB isoform lacking the bHLH and leucine zipper domains, compromises full‐length TFEB function (Park *et al*, 2021). Importantly, post‐translational events constitute an important means of regulating TFEB activity.

In this section, therefore, we focus on the regulatable post‐translational modifications of TFEB (Table 1). We show that, as these post‐translational modifications are sensitive to cellular stresses ranging from nutritional deprivation to infectious burden, they can regulate the subcellular localisation, turnover, dimerisation, and liquid–liquid phase separation (LLPS) of TFEB in a manner that tunes its activity to permit adaptation to such stressors.

**Table 1:** Summary of the known post‐translational modifications of TFEB.

Modification	Enzyme	Modified residue	Effect	References
Phosphorylation	MAP4K3	S3	Recruits TFEB to lysosome	Hsu *et al* (2018)
	mTORC1	S122	Retains TFEB in cytosol	Vega‐Rubin‐de‐Celis *et al* (2017)
		S142	Promotes TFEB nuclear export	Li *et al* (2018), Napolitano *et al* (2018), Settembre *et al* (2012)
		S211	Retains TFEB in cytosol	Martina *et al* (2012), Roczniak‐Ferguson *et al* (2012)
	ERK2	S142	Promotes TFEB nuclear export	Settembre *et al* (2011)
	CDK1/4/6	S142	Promotes TFEB nuclear export	Odle *et al* (2020), Yin *et al* (2020)
	GSK3β	S134	Retains TFEB in cytosol	*Li et al* (2016b)
	GSK3β	S138	Promotes TFEB nuclear export	Li *et al* (2018), *Li et al* (2016b), Napolitano *et al* (2018)
	AKT	S467	Retains TFEB in cytosol	Palmieri *et al* (2017)
	PKCβ	S461 and/or S462, S466, and S468	Reduces TFEB turnover	Ferron *et al* (2013)
	AMPK	S466, S467, and S469	Increases TFEB transcriptional activity	Paquette *et al* (2021)
Acetylation	ACAT1	K91, K103, K116, and K430	Promotes TFEB nuclear translocation	Zhang *et al* (2018)
	Other KATs	K236 and K237	Promotes TFEB nuclear translocation	Li *et al* (2022)
	GCN5	K274 and K279	Disrupts TFEB dimerisation and binding to DNA	W*ang et al* (2020b)
Deacetylation	SIRT1	K116	Increases TFEB transcriptional activity	Bao *et al* (2016), Zheng *et al* (2021)
Ubiquitination	Unknown	K347	Promotes proteasomal degradation of TFEB	Li *et al* (2022)
	STUB1	‐	Promotes proteasomal degradation of TFEB	Sha *et al* (2017)
SUMOylation	‐	K316	Increases TFEB transcriptional activity	Miller *et al* (2005)
Glycosylation	SetA	S195 or S196, T201 or S203, and T208	Promotes TFEB nuclear translocation	Beck *et al* (2020)
Oxidation	‐	C212	Promotes TFEB nuclear translocation	Martina *et al* (2021), Wa*ng et al* (2020a)

### Post‐translational modifications and protein–protein interactions regulating the subcellular localisation of TFEB

As a transcription factor that continually shuttles between the cytosol (where it largely resides at rest) and the nucleus (where it can bind CLEAR motifs within promoters of the genes it transactivates), TFEB translocation to, and export from, the nucleus constitutes principal means of regulating its activity.

#### Regulation of TFEB nuclear translocation by post‐translational modifications

A range of nutritional, lysosomal, ER, mitochondrial, and infectious stresses elicit characteristic post‐translational modifications and protein–protein interactions that influence the nuclear translocation of TFEB.

The main stress‐responsive signalling centre that determines TFEB nuclear translocation is the Ser/Thr kinase, mammalian target of rapamycin (mTOR), which exists in either complex I (mTORC1, containing regulatory‐associated protein of mTOR (RPTOR), mammalian lethal with Sec13 protein 8 (mLST8), and the two inhibitory subunits, DEP domain‐containing mTOR‐interacting protein (DEPTOR) and Pro‐rich Akt substrate of 40 kDa (PRAS40)) or complex II (mTORC2, which also contains rapamycin‐insensitive companion of mTOR (RICTOR), mammalian stress‐activated protein kinase interacting protein (mSIN1), and protein observed with RICTOR1/2 (PROTOR1/2); Dubouloz *et al*, 2005; Sancak *et al*, 2008; Binda *et al*, 2009; Zoncu *et al*, 2011; Martina *et al*, 2012; Roczniak‐Ferguson *et al*, 2012; Settembre *et al*, 2012; Lawrence *et al*, 2018). By phosphorylating effectors, including activation of the ribosomal protein, S6 kinase (S6K), and inhibition of the translational repressor, eIF4E‐binding protein (4E‐BP), mTORC1 triggers anabolic signal transduction pathways that promote cellular and organismal growth. The function of mTORC1 as a master regulator of cell metabolism is contingent on the coincident detection of amino acid concentration via the heterodimeric RagA/B‐RagC/D GTPases, and of growth factor‐driven signalling via the Rheb GTPases (Yang *et al*, 2017; Lawrence *et al*, 2018).

TFEB is a non‐canonical substrate of mTORC1 because it is sensitive to the nucleotide loading status of the Rag, but not the Rheb, GTPases, and thus more dependent on amino acid concentration than on growth factor signalling (Napolitano *et al*, 2020). To phosphorylate cytosolic TFEB, mTORC1 must first be recruited to the lysosome in a manner that depends on the GTP‐bound status of RagA/B (Sancak *et al*, 2008). Under amino acid replete conditions, the late endosomal/lysosomal adaptor, MAPK, and mTOR activator (LAMTOR, “Ragulator”) complex acts as a guanine nucleotide exchange factor (GEF) towards RagA/B, tethering them to the lysosomal membrane, where RagA/B^GTP^ acts as docking sites for RPTOR (Kim *et al*, 2008; Sancak *et al*, 2008, 2010; Nada *et al*, 2009; Bar‐Peled *et al*, 2012). Accordingly, the Leu‐sensitive Sestrin 2 (and, to a lesser extent, Sestrin 1/3; Parmigiani *et al*, 2014) and the Arg‐sensitive cellular arginine sensor for mTORC1 (CASTOR1; Chantranupong *et al*, 2016) associate weakly with the GTPase‐accelerating proteins (GAPs) towards Rags (GATOR) subcomplex 2 (GATOR2), which represses the GAP function of GATOR1 towards RagA/B, thereby ensuring it remains bound to GTP (Parmigiani *et al*, 2014; Chantranupong *et al*, 2016). Likewise, the methyl donor, *S*‐adenosylmethionine (SAM), disrupts activation of GATOR1 by S‐adenosylmethionine sensor upstream of mTORC1 (SAMTOR) (Gu *et al*, 2017; Tang *et al*, 2022). Furthermore, high‐cholesterol concentrations stimulate the GPCR‐like protein, lysosomal cholesterol signalling (LYCHOS), to sequester GATOR1 (Shin *et al*, 2022). Along with the cholesterol‐ and Arg‐responsive lysosomal multipass transmembrane protein, SLC38A9, possibly catalysing the formation of RagA^GTP^, this enables lysosomal recruitment of mTORC1 under conditions of amino acid and cholesterol sufficiency (Castellano *et al*, 2017; Shen & Sabatini, 2018).

Having been recruited to the lysosome, ragulator‐mTORC1 associates with, and acts upon, its non‐canonical substrates, including TFEB, in a manner that depends on the GDP‐bound status of RagC/D (Wada *et al*, 2016; Lawrence *et al*, 2019; Gollwitzer *et al*, 2022; Kimura *et al*, 2022; Cui *et al*, 2023). As such, multiple other nutrient‐sensitive proteins regulate mTORC1‐dependent phosphorylation of TFEB by modulating the nucleotide‐loading status of RagC/D. The tumour suppressor protein, folliculin (FLCN), heterodimerises with folliculin‐interacting proteins 1 and 2 (FNIP1 and FNIP2) to form a complex that cycles between the lysosome, where it forms a catalytically inactive lysosomal folliculin complex (LFC) with regulator and inactive RagA/B^GDP^‐RagC/D^GTP^ heterodimer (Lawrence *et al*, 2019), and the cytosol, where it is catalytically active as its essential Arg is no longer displaced by the RagC nucleotide (Petit *et al*, 2013; Tsun *et al*, 2013; Meng & Ferguson, 2018). In the presence of amino acids, GATOR1‐induced formation of RagA/B^GDP^ is suppressed, and the cytosolic tail of SLC38A9 destabilises the LFC (Fromm *et al*, 2020), promoting LFC disassembly and thus cytosolic FLCN‐FNIP1/2‐mediated RagC/D^GDP^ generation (Tsun *et al*, 2013; Meng & Ferguson, 2018). Moreover, leucyl tRNA synthetase (LRS) exhibits GAP activity towards RagD (Kim *et al*, 2017), in a fashion tightly coordinated with the actions of sestrin 2 (Lee *et al*, 2018).

Therefore, under amino acid replete conditions, S3 in the N‐terminus of TFEB is phosphorylated by mitogen‐activated protein 4 kinase 3 (MAP4K3) (Hsu *et al*, 2018), enabling recruitment by RagC/D^GDP^ to lysosomal mTORC1, which phosphorylates it at several sites, including S122, S142 (also targeted by the nutrient‐responsive MAPK1 (ERK2)) and S211 (Dephoure *et al*, 2008; Huttlin *et al*, 2010; Settembre *et al*, 2011, 2012; Martina *et al*, 2012; Roczniak‐Ferguson *et al*, 2012; Vega‐Rubin‐de‐Celis *et al*, 2017). Phosphorylation of S211, in turn, masks the nuclear localisation signal (NLS) and creates a binding site for the cytosolic chaperone 14‐3‐3, which sequesters TFEB in the cytosol (Martina *et al*, 2012; Roczniak‐Ferguson *et al*, 2012; Xu *et al*, 2019) in a manner strengthened by glycogen synthase kinase‐3 (beta) (GSK3(beta))‐mediated phosphorylation of Ser134 and Ser138 (*Li et al*, 2016b). Interestingly, during the G1/S transition of the cell cycle, cyclin‐dependent kinases (CDKs) 4 and 6 can also bind and phosphorylate TFEB on mTORC1‐targeted sites, suppressing TFEB upon commitment to cell division (Yin *et al*, 2020), during which CDK1 also takes over from the otherwise inactive mTORC1 to ensure TFEB remains phosphorylated (Odle *et al*, 2020).

By contrast, in amino acid starvation, GATOR1 function predominates and FLCN‐FNIP1/2 no longer act towards GAPs towards RagC/D, such that RagA/B^GDP^‐RagC/D^GTP^ becomes the dominant configuration of the Rag‐GTPases, preventing the lysosomal assembly of mTORC1 and its recruitment of TFEB, respectively. Moreover, amino acid starvation promotes Ca^2+^ efflux through the lysosomal channel, mucolipin 1 (MCOLN1), which creates a perilysosomal Ca^2+^ microdomain that activates the phosphatase calcineurin (CaN), which can sequentially dephosphorylate TFEB at mTORC1‐targeted sites, thereby unmasking its NLS and dissociating it from 14‐3‐3 (Medina *et al*, 2015; Vega‐Rubin‐de‐Celis *et al*, 2017). Moreover, prolonged ER stress can elicit the integrated stress response (ISR), stimulating the eukaryotic translation initiation factor 2 (eIF2) kinase, PKR‐like ER kinase (PERK), to activate CaN and thus promote nuclear translocation of TFEB (Martina *et al*, 2016). Notably, MCOLN1 activity is sensitive to oxidative stress, such that reactive oxygen species (ROS) can directly stimulate MCOLN1‐mediated Ca^2+^ efflux, CaN activation, and TFEB nuclear translocation (Zhang *et al*, 2016). In fact, oxidative stress can also activate another phosphatase, protein phosphatase 2A (PP2A), which dephosphorylates TFEB on S109, S114, S122, and S211 (Martina & Puertollano, 2018). Oxidative stress also promotes the oxidation of Cys212 within TFEB, an event sufficient to induce its nuclear translocation within 8 min by facilitating disulphide bond formation with other TFEB molecules, such that homo‐oligomerisation reduces mTORC1 access to S122, S142, and S211 (Wa*ng et al*, 2020a; Martina *et al*, 2021). Likewise, infectious burden stimulates the glucosyltransferase, SetA, to glucosylate S195, S196, T201, S203, and T208, disrupting the binding of TFEB to 14‐3‐3, and thus promoting its nuclear localisation (Beck *et al*, 2020). Moreover, transcriptional stress‐induced acetylation of some of the Lys residues in TFEB may promote its nuclear translocation independent of changes in canonical mTORC1 activity (Zhang *et al*, 2018). Indeed, histone deacetylases (HDACs) and lysine acetyltransferases (KATs) acetylate K236 and K237 within the NLS of TFEB, promoting its dissociation from 14‐3‐3, thereby driving its translocation to the nucleus (Li *et al*, 2022).

**Figure 2:**
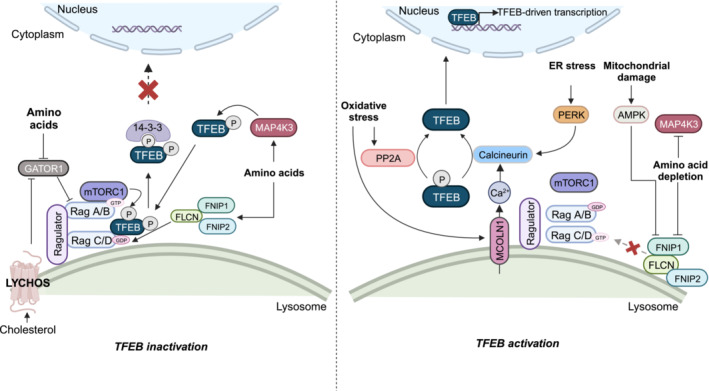
Phosphorylation as a stress‐sensitive regulator of TFEB subcellular localisation Left: In amino acid and cholesterol‐replete conditions, lysosomal mTORC1 recruits and phosphorylates TFEB, which promotes its cytosolic sequestration. Right: Nutrient deprivation, lysosomal, and mitochondrial stresses converge on diminished mTOR activity towards, and on the removal of phosphate groups from, TFEB, promoting its nuclear translocation. Created with BioRender.com.

Furthermore, in response to mitochondrial damage, adenosine monophosphate‐activated kinase (AMPK) phosphorylates FNIP1 to suppress the GAP activity of FLCN‐FNIP1/2 towards RagC/D, which are thus retained in the RagC/D^GTP^‐bound form, which chaperones the N‐terminus of TFEB off the lysosome such that it is no longer phosphorylated by mTORC1, and translocates to the nucleus (Malik *et al*, 2023) (Fig 2). Likewise, in response to lysosomal damage, membrane compartments conjugated to GABARAP sequester the LC3‐interacting motif (LIR) of FLCN‐FNIP1/2 to prevent RagC/D^GDP^ formation and mTORC1‐mediated TFEB phosphorylation, while LC3B also accumulates on the lysosomal membrane, where its lipidated form, LC3B‐II, activates MCOLN1 to activate CaN and drive TFEB into the nucleus (Nakamura *et al*, 2020; Goodwin *et al*, 2021).

Finally, although much less sensitive to growth factor signalling than nutritional status and organelle damage, TFEB nuclear translocation can be influenced by Wnt, which regulates the (beta)‐catenin destruction complex (Axin, anaphase‐promoting complex (APC), and GSK3(beta)). Wnt‐mediated poly(ADP)‐ribosylation (PARsylation) of TFEB prevents it from interacting with cytosolic Axin and APC, thereby promoting translocation of TFEB to the nucleus, where it interacts with a distinct (beta)‐catenin complex (Kim *et al*, 2021).

#### Regulation of TFEB nuclear export by post‐translational modifications

**Figure 3:**
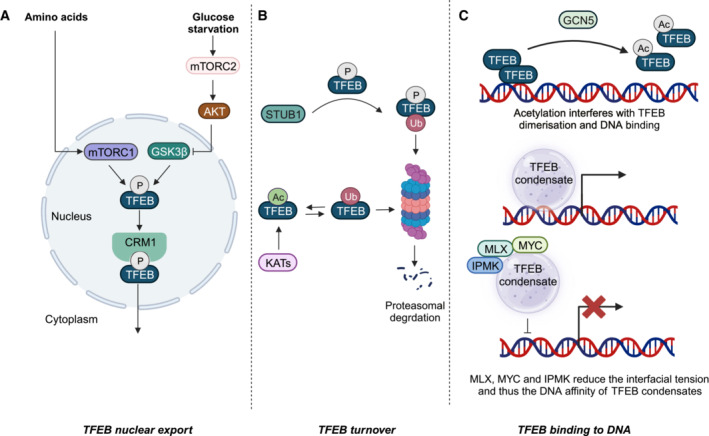
Post‐translational regulation of TFEB nuclear export, turnover, and DNA‐binding capacity (A) Deprivation of amino acids and glucose impairs the nuclear export of TFEB by reducing its phosphorylation. (B) Amino acid starvation promotes the association of phosphorylated TFEB with the E3 ubiquitin ligase, STUB1. The acetylation of one amino acid within TFEB impairs its ubiquitination and proteasomal degradation. (C) Acetylation can disrupt TFEB homo‐ and heterodimerisation. A subset of the TFEB interactome reduces the interfacial tension of TFEB condensates, decreasing their affinity for DNA. Created with BioRender.com.

A range of nutritional and infectious stresses elicit characteristic post‐translational modifications and protein–protein interactions that influence the nuclear export of TFEB (Fig 3).

The phosphorylation of the TFEB S142 residue by mTORC1 and/or ERK2 in amino acid replete conditions primes GSK3(beta) to phosphorylate S138, whereupon the coincidence of both phosphorylated residues activates the nuclear export signal (NES) between residues 140 and 150, such that it can interact with the nuclear export protein, chromosomal region maintenance 1 (CRM1; *Li et al*, 2016b, 2018; Napolitano *et al*, 2018; Silvestrini *et al*, 2018). By contrast, glucose starvation activates mTORC2 and protein kinase B (AKT), which inactivates GSK3(beta) and thus prevents nuclear export of TFEB, even when it is re‐phosphorylated at S142 (Li *et al*, 2018; Fig 3A). Given that the nuclear export of re‐phosphorylated TFEB constitutes a rate‐limiting step in determining its subcellular localisation, the ability of the NES within TFEB to integrate carbon and nitrogen availability is crucial to the capacity of TFEB to dynamically respond to nutritional deprivation and refeeding (Li *et al*, 2018; Napolitano *et al*, 2018; Zhitomirsky *et al*, 2018). In a similar fashion, during bacterial infection, SetA glucosylates TFEB at S138 to compete with GSK3(beta)‐mediated phosphorylation, preventing nuclear export (Beck *et al*, 2020).

### Post‐translational modifications and protein–protein interactions regulating the turnover of TFEB

As a transcription factor that largely resides in the cytosol in the absence of significant nutritional and intracellular stress, TFEB is subject to continued proteasomal degradation, which constitutes the principal means of regulating its turnover (Fig 3B).

In amino acid starvation, the E3 ubiquitin ligase, STIP1 homology, and U‐box containing protein 1 (STUB1) interact with phosphorylated S142 and S211 on TFEB, promoting its ubiquitination, and thus preferentially shortening the half‐life of transcriptionally inactive TFEB (Sha *et al*, 2017). Interestingly, receptor activator of nuclear factor (kappa)‐B ligand (RANKL) signalling can promote PKC(beta)‐mediated phosphorylation of C‐terminal S462, S463, S467, and S469, which, in turn, increases the stability of cytosolic TFEB (Ferron *et al*, 2013). Furthermore, transcriptional stress‐induced acetylation of TFEB at K347 suppresses its K48‐linked polyubiquitination at that site (Li *et al*, 2022), raising the possibility that multiple moieties can influence proteasomal degradation of TFEB in response to stress signals.

### Post‐translational modifications and protein–protein interactions regulating the DNA‐binding capacity of TFEB

As a bHLH transcription factor, TFEB binding to DNA depends on its ability to: (i) homo‐ and heterodimerise with other MiT/TFE family members; and (ii) undergo LLPS to form condensates to which the machinery of transcription can be recruited (Fig 3C).

The acetyltransferase, KAT2A (GCN5), acetylates K274 and K279 of TFEB, disrupting its homo‐ and heterodimerisation, and thus reduces its affinity for the CLEAR motif‐containing promoters of its target genes (Wa*ng et al*, 2020b). However, the post‐translational addition of some moieties, such as phosphates to the C‐terminus by AMPK in response to amino acid starvation (Paquette *et al*, 2021), or small ubiquitin‐like modifiers (SUMOs) to K316 (Miller *et al*, 2005), respectively, promote and suppress the transcriptional activity of TFEB independently of its subcellular localisation by as‐yet unclear mechanisms. Nuclear TFEB undergoes LLPS to recruit the mediator complex to membraneless compartments in which the mRNAs of its target genes can be formed. A subset of the nuclear TFEB interactome, including Max‐like protein X (MLX), MYC (Wang *et al*, 2023), and inositol polyphosphate multikinase (IPMK; Chen *et al*, 2020), can act as surfactants, reducing the interfacial tension of TFEB liquid condensates to reduce its affinity for the CLEAR motifs in the promoters of its target genes, thereby limiting its activity (Fig 3C). It remains to be seen whether interactors of TFEB's disordered Gln‐rich N‐terminus can also mediate or disrupt its LLPS (Zhang *et al*, 2015).

## The functional consequences of TFEB (Dys)regulation: from physiology to pathology

### TFEB as a master regulator of organelle biogenesis

Although organelles can be created *de novo*, biogenesis predominantly proceeds from existing populations. TFEB acts as a master regulator of lysosomal biogenesis by coordinating the transcriptional upregulation of lysosomal membrane proteins and lysosomal hydrolases (Sardiello *et al*, 2009; Palmieri *et al*, 2011), biosynthesis of which, when coordinated with endosome–lysosome trafficking, promotes endocytic (ELR), autophagic (ALR), and phagocytic (PLR) lysosomal reformation (Yang & Wang, 2021).

**Figure 4:**
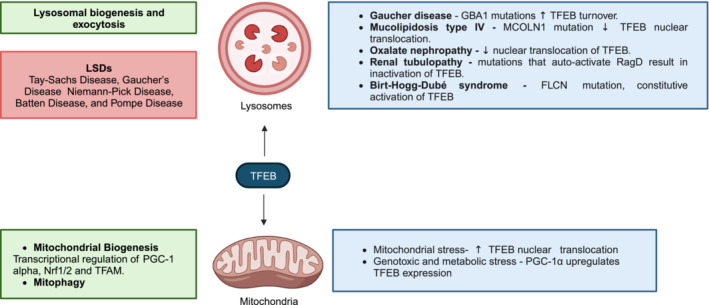
TFEB as a regulator of organelle biogenesis and quality control TFEB acts as the master regulator of lysosomal biogenesis and lysosomal exocytosis, while also inducing the transcription of the master regulator of mitochondrial biogenesis. Key: green—physiological role of TFEB; red—diseases where TFEB activity is postulated to contribute; blue—alterations in TFEB function reflecting the intracellular stresses imposed by pathological conditions. Created with BioRender.com.

By contrast, TFEB regulates mitochondrial protein import, as well as fusion and fission, by transcriptionally upregulating a transcriptional coactivator, peroxisome proliferator‐activated receptor gamma (PPARG) coactivator 1‐(alpha) (PGC‐1(alpha)), which then acts as the master regulator of mitochondrial biogenesis (Settembre *et al*, 2013; Fig 4).

#### TFEB‐induced lysosomal biogenesis and lysosomal exocytosis

Lysosomes are membrane‐bound organelles that, through the vacuolar H^+^‐ATPase (v‐ATPase), provide an acidic compartment to optimise the degradative activity of 60 hydrolytic enzymes within their lumina, including nucleases, proteases, (phospho)lipases, and phosphatases. Representing the endpoint of catabolic pathways, the lysosome interacts with the cellular signalling machinery to limit intracellular nutrient oscillations as membrane‐spanning permeases export the metabolites formed by intralysosomal hydrolysis into the cytosol (Rong *et al*, 2011; Verdon *et al*, 2017; Wyant *et al*, 2018, p. 201), wherein some are immediately utilised, and others are stored or used as buffers (Russnak *et al*, 2001; Li & Kane, 2009; Abu‐Remaileh *et al*, 2017; Verdon *et al*, 2017). Moreover, the lysosome can fuse with the plasma membrane either to discharge its contents to the extracellular fluid (lysosomal exocytosis) or to repair damage to the phospholipid bilayer.

As a master transcriptional regulator of lysosomal biogenesis and lysosomal exocytosis, TFEB plays a crucial role in facilitating hydrolysis of intralysosomal contents and nutritional rebalance. Lysosomal stressors—by eliciting the post‐translational addition of a characteristic set of moieties to, and the interaction of a specific set of proteins with, TFEB—alter TFEB activity in a manner that promotes cellular adaptation to such stresses.

In pathological states, constant lysosomal stress may saturate the capacity of post‐translational modifications and protein–protein interactions to further increase compensatory TFEB activity, possibly sustaining disease. The impaired degradation of intralysosomal material underlying lysosomal storage disorders (LSDs) generates a propensity for lysosomal stress, establishing these diseases as a model for elucidating the cytoprotective role of TFEB‐induced lysosomal biogenesis and exocytosis.

The LSDs are typically caused by loss of function of the lysosomal enzymes that hydrolyse gangliosides (as in Tay‐Sachs disease), sphingolipids (as in Gaucher's and Niemann–Pick diseases), glycerophosphodiesters (as in Batten disease), glycosaminoglycans (as in mucopolysaccharidoses and multiple sulfatase deficiency (MSD)), and glycogen (as in Pompe disease). Also implicated are membrane transport proteins, such as N‐acetylglucosamine‐1‐phosphate transferase (as in mucolipidoses types II and IIIA). All such defects in lysosomal enzymes and transporters drive a propensity for the affected cell to accumulate the corresponding substrates, which form cytotoxic aggregates. The intracellular environment archetypal of LSDs thus selects for endogenous signalling pathways that can promote lysosomal biogenesis and lysosomal exocytosis. By increasing the number of lysosomes that dock and fuse to the plasma membrane, TFEB is a key initiator of one such pathway (Medina *et al*, 2011). Indeed, overexpression of TFEB rescues the cytotoxicity arising from the accumulation of toxic lipid, mucopolysaccharide, and glycogen aggregates in mouse models of MSD, mucopolysaccharidosis type IIIA, and Pompe disease, respectively (Spampanato *et al*, 2013). Furthermore, the ability of genistein and 2‐hydroxypropyl‐(beta)‐cyclodextrin to alleviate pathology in mucopolysaccharidoses and Niemann–Pick Type C depends at least partly on TFEB (Moskot *et al*, 2014; Song *et al*, 2014), and direct pharmacological activation of TFEB by limiting its Akt‐mediated phosphorylation reduces aggregation of the ceroid lipopigment that typically accumulates in Batten disease (Palmieri *et al*, 2017). Likewise, the ability of weakly basic lysosomotropic chemotherapeutics, such as tamoxifen (Soldati *et al*, 2021), and selective serotonin reuptake inhibitors (SSRIs), such as fluoxetine (Capuozzo *et al*, 2022), to activate TFEB‐mediated lysosomal exocytosis suggests that they may be candidates for repurposing in the treatment of LSDs.

However, the intracellular environment characteristic of LSDs establishes a set of post‐translational modifications on TFEB that either impair its role in lysosomal biogenesis and lysosomal exocytosis, thereby suppressing its cytoprotective function, or that activate it in a compensatory manner. For instance, in neurons carrying the glucosylceramidase beta 1 (GBA1) mutations, characteristic of Gaucher's disease, mutant glucocerebrosidase considerably increases TFEB turnover, thereby impairing lysosomal biogenesis and worsening pathology (Awad *et al*, 2015). Moreover, loss‐of‐function mutations in the lysosomal membrane protein and CLEAR network gene, MCOLN1, typical of mucolipidosis type IV abrogate TFEB‐induced aggregate clearance (Medina *et al*, 2011). This arises, in part, from the diminished ability of TFEB to increase its own activity within the setting of LSDs; even when MCOLN1 is transcriptionally upregulated by TFEB, it is no longer able to release lysosomal Ca^2+^ and activate CaN, thereby preventing dephosphorylation and nuclear translocation of TFEB (Fig 4).

Given the propensity of LSDs to impair the capacity of post‐translational modifications and protein–protein interactions to increase TFEB activity, exogenous delivery or direct stimulation of TFEB may be a suitable therapeutic option (Song *et al*, 2013), especially when paired with recombinant therapeutic enzymes (Awad *et al*, 2015), as TFEB can upregulate the mutated proteins with residual enzymatic function.

Nephropathies also provide a paradigm for understanding how lysosomal stress can introduce a set of post‐translational modifications that impair or compensatorily activate TFEB‐mediated lysosomal biogenesis and exocytosis. Indeed, a subset of renal tubulopathy is caused by mutations that auto‐activate RagD, thereby promoting TFEB recruitment to, and phosphorylation by, lysosomal mTORC1, such that it can no longer induce lysosomal biogenesis and lysosomal exocytosis in response to lysosomal damage (Sambri *et al*, 2023).

By contrast, patients with oxalate nephropathy, a disease characterised by the build‐up of calcium oxalate crystals within the lysosomes of proximal tubule epithelial cells (Mulay & Anders, 2017), exhibit enhanced nuclear translocation of TFEB, raising the possibility that the intracellular environment augments the ability of damaged lysosomes that accumulate LC3B to stimulate MCOLN1 and thus dephosphorylate TFEB (Nakamura *et al*, 2020; Fig 4).

#### TFEB‐induced mitochondrial biogenesis

Mitochondria are double‐membrane‐bound organelles that, by providing a series of protein complexes and generating a mitochondrial membrane potential (MMP), optimise the oxidative phosphorylation of metabolic intermediates by the electron transport chain to synthesise ATP.

Owing to its cooperation with the master regulator of mitochondrial biogenesis, PGC‐1(alpha), TFEB plays a key role in mitochondrial function. Indeed, mitochondrial stress activates AMPK, which phosphorylates FNIP1 to reduce RagC/D^GDP^ formation and promote the nuclear translocation of TFEB, which can transcriptionally upregulate PGC‐1(alpha) (Malik *et al*, 2023). In turn, PGC‐1(alpha) can bind within the promoter of the gene‐encoding TFEB (Tsunemi *et al*, 2012), establishing a feedback loop sustaining the rise in its expression and activity, and thereby promoting mitochondrial biogenesis to adapt to genotoxic (Lynch *et al*, 2019) and metabolic stresses. However, TFEB can also regulate mitochondrial biogenesis independently of PGC‐1(alpha). TFEB transcriptionally upregulates nuclear respiratory factors 1 and 2 (Nrf1/2) and mitochondrial transcription factor A (TFAM), thereby promoting an increase in mitochondrial volume and density (Mansueto *et al*, 2017), as well as in mitochondrial DNA (mtDNA) synthesis (Farge & Falkenberg, 2019) and peroxidase (Eun *et al*, 2018) transcription (Fig 4). Moreover, TFEB reduces mitochondrial division (Santin *et al*, 2016) and, by directly regulating the expression of genes encoding glucose transporters and glycolytic enzymes, optimises the mitochondrial utilisation of metabolic substrates for oxidative phosphorylation (Mansueto *et al*, 2017).

### TFEB as a master regulator of macro‐autophagy

Autophagy is the sequestration of intracellular and extracellular material to lysosomes for their degradation. There are three types of autophagy: (i) macro‐autophagy, which relies on the formation of double‐membraned vesicles (autophagosomes) to deliver extra‐ or intracellular material to the lysosome; (ii) chaperone‐mediated autophagy (CMA), in which proteins assist the entry of material into lysosomes; and (iii) micro‐autophagy, whereby cargo is internalised through direct invagination of the lysosomal membrane. In macro‐autophagy, the biogenesis and expansion of membranous precursors to the final autophagosome are regulated by a series of ubiquitin‐like conjugations using autophagy‐related gene (ATG) proteins.

Although TFEB can drive macro‐autophagy by inducing lysosomal biogenesis as a master transcriptional regulator of the ATGs, TFEB also plays a crucial lysosome‐independent role in facilitating autophagosome biogenesis and autophagic substrate delivery to the lysosome.

**Figure 5:**
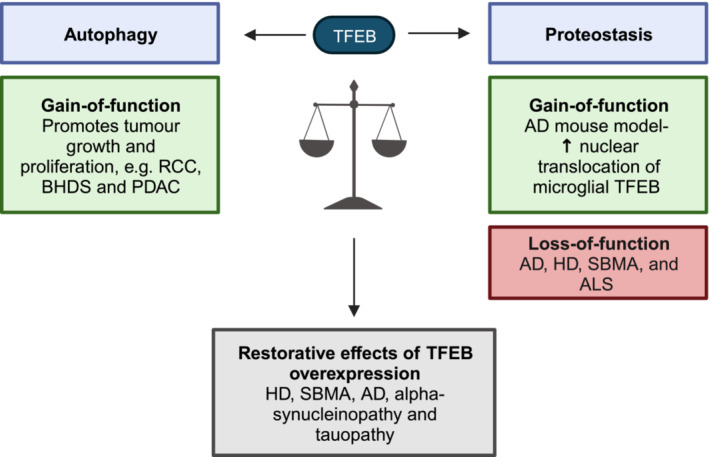
Balancing autophagy and proteostasis via TFEB: shaping disease outcomes from tumorigenesis to neurodegeneration Despite the inhibitory role of macro‐autophagy on tumorigenesis, TFEB acts as an oncogene in translocation renal cell carcinoma (tRCC) and Birt–Hogg–Dubé syndrome (BHDS). As expected from the contribution of macro‐autophagy to metabolic homeostasis, TFEB promotes the proliferation of established tumours in RCC, BHDS, and pancreatic ductal adenocarcinoma (PDAC). Neurodegenerative diseases, such as Huntington's disease (HD), Alzheimer's disease (AD), spinal‐bulbar muscular atrophy (SBMA), (alpha)‐synucleinopathy, and tauopathy, depress neuronal, and elevate microglial, TFEB activity, such that overexpression or activation of TFEB can rescue loss of susceptible neuronal populations. See review text for more information. Created with BioRender.com.

By eliciting the post‐translational addition of a characteristic set of moieties to TFEB, and the interaction of a specific set of proteins with it, nutrient starvation and hypoxia alter TFEB activity in a manner that promotes cellular adaptation to such stresses. As such, the pathological role of TFEB‐mediated induction of macro‐autophagy varies with the disease‐specific selection pressures that arise within the affected cell(s) (Fig 5).

#### TFEB‐induced macro‐autophagy in proteostasis

The cytosolic accumulation of neurotoxic protein aggregates is a feature common to several neurodegenerative diseases, including those caused by polyglutamine (polyQ) tract expansions, such as Huntington's disease and some spinocerebellar ataxias, (alpha)‐synucleinopathy (seen in Parkinson's disease), TDP‐43 proteinopathy (seen in ALS and some dementias), and tauopathies (seen in Alzheimer's disease and dementias). Under physiological conditions, macro‐autophagy can deliver oligomeric proteins to the lysosome for degradation. Unsurprisingly, therefore, patients with some hereditary neurodegenerative and neurodevelopmental diseases have loss‐of‐function mutations and polymorphisms in core ATGs, as well as those encoding proteins involved in the trafficking, maturation, and lysosomal fusion of autophagosomes. Moreover, the prodromal phase of sporadic neurodegenerative disease is characterised by the coincidence of age‐related neuroinflammation and impairment of macro‐autophagy and CMA (Sala *et al*, 2016).

TFEB overexpression in models of Huntington's disease and spinal and bulbar muscular atrophy (SBMA, “Kennedy's Disease”) can increase autophagic clearance of polyQ‐expanded mutant huntingtin (mHtt; Sardiello *et al*, 2009; La Spada, 2012; Tsunemi *et al*, 2012) or androgen receptor (mAR; Cortes *et al*, 2014), limiting their accumulation into toxic aggregates. In dopaminergic neurons from the midbrains of (alpha)‐synucleinopathy models, TFEB overexpression or post‐translational hyperactivation downstream of mTORC1 rescues the aggregation of (alpha)‐synuclein, halting the progression of neuronal loss (Decressac *et al*, 2013). Likewise, TFEB overexpression can increase the clearance of hyperphosphorylated and misfolded tau without influencing tau phosphorylation directly (Ramsden, 2005; SantaCruz *et al*, 2005; Polito *et al*, 2014), implicating upregulation of macro‐autophagy as its mechanistic basis (Chauhan *et al*, 2015). Moreover, transactivation of TFEB transcriptional targets may limit the accumulation of beta‐amyloid either by increasing autophagic degradation of the amyloid precursor protein (APP), or by increasing (Parr *et al*, 2012) the clearance of toxic amyloid (beta) aggregates in some (Xiao *et al*, 2015), but not all (Oakley *et al*, 2006; Polito *et al*, 2014), mouse models of Alzheimer's disease. TFEB can also affect glial cell clearance of toxic protein aggregates; astrocytic TFEB overexpression can accelerate the degradation of extracellular amyloid (beta) by neuronal autophagy in mouse models of Alzheimer's disease (Xiao *et al*, 2014).

The intracellular environment characteristic of susceptible neurons may result in a set of post‐translational modifications and protein–protein interactions that impair the ability of TFEB to induce macro‐autophagy, and thereby suppress its otherwise neuroprotective function. For instance, (alpha)‐synuclein is structurally homologous to 14‐3‐3 (Ostrerova *et al*, 1999; Perez *et al*, 2002), such that it can sustain TFEB sequestration within the cytosol (Decressac *et al*, 2013), where it remains transcriptionally inactive. Furthermore, loss of function of GBA1, mutations of which are the most common genetic cause of sporadic Parkinson's disease, increases TFEB turnover, further impairing transcriptional induction of macro‐autophagy and exacerbating (alpha)‐synucleinopathy (Awad *et al*, 2015). Moreover, neurons from the brains of patients with Alzheimer's disease contain elevated levels of acid sphingomyelinase, which impairs the TFEB‐mediated upregulation of ATGs and induction of lysosomal biogenesis (Lee *et al*, 2014). In neurons from models of both Huntington's disease and Kennedy's disease, expanded polyQ tracts in mHtt (Yang *et al*, 2023) or mAR (Cortes *et al*, 2014) are able to bind and sequester the disordered Gln‐rich N‐terminus of TFEB, and disrupt its ability to upregulate the ATGs (Tsunemi *et al*, 2012), perhaps by impairing phase separation (Wang *et al*, 2022). However, skeletal muscle from SBMA transgenic mice (Sopher *et al*, 2004; Yu, 2006) exhibits significant nuclear translocation of TFEB (Chua *et al*, 2014), implying a tissue‐specific pathological mechanism. Interestingly, although one of the causes of ALS, hexanucleotide repeat expansions in C9orf72, can similarly sequester TFEB within the cytosol (Cunningham *et al*, 2020) partly through Rag‐GTPase dysregulation (Ji *et al*, 2020), depletion of another component of ALS pathogenesis, TDP‐43, downregulates RPTOR and thus reduces mTORC1‐mediated phosphorylation of TFEB, promoting its nuclear translocation and transactivation of ATG expression.

In contrast to neurons vulnerable to degeneration, glia in their microenvironment may generate a series of tissue‐specific post‐translational modifications that promote TFEB gain of function. Indeed, in the prodrome of transgenic mouse models of Alzheimer's disease, fibrillar amyloid (beta) stimulates the nuclear translocation of microglial TFEB, in part through sirtuin 1 (SIRT1)‐mediated deacetylation of TFEB at K116. The consequent upregulation of the ATGs enables macro‐autophagic clearance of APP and fibrillar amyloid (beta; Bao *et al*, 2016; Zheng *et al*, 2021). Likewise, astrocytic overexpression of TFEB can also rescue tauopathy in similar disease models (Martini‐Stoica *et al*, 2018; Fig 5).

#### TFEB‐induced macro‐autophagy in organelle quality control

Macro‐autophagy can selectively deliver organelles, ranging from nuclei (nucleophagy) to mitochondria (mitophagy) and ER (ER‐phagy), to the lysosome for degradation (organellophagy). Although organellophagy relies on ATG proteins, it can be regulated independently from other forms of macro‐autophagy.

Cancer cells and cardiomyocytes are predisposed to oxidative stress, thus selecting pathways that ensure mitochondrial quality control, including mitophagy (Guo *et al*, 2013), and these pathological states thus provide a paradigm for understanding the cytoprotective role of TFEB‐induced activation of mitophagy. In established tumours, there is a switch to Warburg metabolism and re‐routing of glycolysis intermediates into the pentose phosphate pathway. Indeed, mitochondrial stress and MMP depolarisation induce PINK1‐mediated recruitment of the E3 ubiquitin ligase, PARKIN, from the cytosol to the outer mitochondrial membrane, where it ubiquitinates proteins including mitofusins and Miro1 to initiate autophagosome formation and selective elimination of damaged mitochondria (Narendra *et al*, 2012). Even in the background of intact macro‐autophagy, loss of function of the mitophagy receptors, BNIP3 or NIX, can promote breast and pancreatic tumorigenesis (Chourasia *et al*, 2015; Humpton *et al*, 2019). PINK1, PARKIN, ATG5, and ATG9A can induce nuclear translocation of TFEB (Nezich *et al*, 2015), which facilitates mitophagic clearance of impaired mitochondria (Ma *et al*, 2015; Nezich *et al*, 2015). TFEB‐induced mitophagy accounts, in part, for the cardioprotective function of cobalt protoporphyrin IX (Unuma *et al*, 2013).

By contrast, the intracellular environment in neurons susceptible to degeneration creates a set of post‐translational modifications on, and protein interactions with, TFEB that impair its role in mitophagy. For instance, the parkin Q311X mutation present in a subset of Parkinson's disease patients reduces the turnover of parkin‐interacting substrate (PARIS), relieving its transcriptional repression of PGC1‐(alpha), and thus downregulating TFEB and suppressing TFEB‐facilitated mitophagy (Siddiqui *et al*, 2015). As such, in dopaminergic neurons characterised by mitochondrial compromise, pharmacological inhibition of mTORC1 to post‐translationally hyperactivate TFEB can restore viability (Siddiqui *et al*, 2015).

Constantly proliferating in response to mechanical cues, chondrocytes provide a paradigm for understanding the cytoprotective role of TFEB‐mediated induction of ER‐phagy. Although the ISR promotes TFEB nuclear translocation independently of mTORC1 in order to increase resistance to the apoptosis that would otherwise result from prolonged ER stress (Martina *et al*, 2016), TFEB can also directly regulate ER‐phagy. For instance, during skeletal growth, both nutrient starvation and FGF signalling converge on mTORC1 inhibition, whereupon TFEB translocates to the nucleus and upregulates transcription of the ER‐phagy receptor, FAM134B, thereby facilitating protein secretion in chondrocytes (Cinque *et al*, 2020).

#### Macro‐autophagy‐dependent and ‐independent roles of TFEB in genomic and metabolic homeostasis

Predisposed to genomic instability, nutritional, and oxidative stress in a stage‐dependent manner, cancer cell formation (tumorigenesis) and subsequent survival provide a paradigm for understanding the diverse pathogenic roles of TFEB‐induced upregulation of the ATGs and other CLEAR network genes.

At the stage of gastrointestinal and hepatic tumorigenesis, cancer cell survival seems to depend, in part, on loss of function of ATGs, including ATG2B, ATG5, ATG7 (Takamura *et al*, 2011), ATG9B, and ATG12 (Kang *et al*, 2009; Frangež *et al*, 2021), suggesting that macro‐autophagy suppresses the ability of genomic instability to promote oncogenic transformation. By contrast, once this barrier has been overcome and oncogenic transformation has occurred, the rapid proliferation and aberrant growth factor signalling in the tumour create a dependency on macro‐autophagy (Guo *et al*, 2011; Rosenfeldt *et al*, 2013; Yang *et al*, 2014) to provide an endogenous source of nutrition to mitigate the insufficiency of external nutrients (Bhatt *et al*, 2019). Finally, despite promoting the migration (Lock *et al*, 2014; Sharifi *et al*, 2016) and epithelial‐to‐mesenchymal transition (Wei *et al*, 2014) of cancer cells, ATG proteins stage‐specifically inhibit disseminated cancer cells' emergence from dormancy (Morris *et al*, 1993; La Belle Flynn *et al*, 2019) and outgrowth into a lethal metastasis (“metastatic colonisation”; Lambert *et al*, 2017) (Aqbi *et al*, 2018; Marsh *et al*, 2020).

As such, the effects of TFEB‐mediated upregulation of ATGs depend acutely on the stage of tumour progression.

Interestingly, 1–5% of adult, and up to 40% of paediatric, renal cell carcinomas (tRCCs) are caused by chromosomal translocation of MiT/TFE family members, including TFEB (Linehan *et al*, 2010; Malouf *et al*, 2014). These translocation events fuse the bHLH‐, DNA‐binding, and NLS‐containing sequences within the C‐terminus of TFEB to the N‐termini of proteins encoded by genes with constitutively active promoters, thereby preserving TFEB's open reading frame and amplifying its transcriptional expression (Kuiper, 2003). Moreover, TFEB upregulation may contribute to tumour formation in sporadic RCC (Durinck *et al*, 2015; Gupta *et al*, 2017), and depletion of TFEB is sufficient to prevent tumorigenesis in xenografts derived from renal tumours in patients with Birt–Hogg–Dubé syndrome (BHDS; Di Malta *et al*, 2023). In fact, BHDS is characterised by loss‐of‐function mutations in FLCN, preventing RagC/D^GDP^ formation and thus recruitment of TFEB to, and thus post‐translational phosphorylation by, lysosomal mTORC1, driving TFEB nuclear translocation and hyperactivation (Napolitano *et al*, 2020). This suggests that TFEB drives tumorigenesis despite inducing the otherwise tumour‐suppressive macro‐autophagy programme (Fig 5).

Indeed, the ability of TFEB to act as an oncogene, when transcriptionally amplified or post‐translationally hyperactivated, likely depends on its regulation of the cell cycle rather than of macro‐autophagy. In fact, TFEB is not only phosphorylated but can also transcriptionally upregulate CDK4/6 to promote the G1/S transition and commitment to cell division (Doronzo *et al*, 2019). Furthermore, TFEB can control the transcription of p21, enabling cellular adaptation to genotoxic stresses, and thus raising the possibility that it promotes survival in the face of genomic instability (Pisonero‐Vaquero *et al*, 2020). Moreover, TFEB may promote tumorigenesis due to differences between its canonical targets and its transcriptional signature when amplified or fused. For instance, tRCC is characterised by much higher NRF2 activity than other RCC subtypes, and, in the context of papillary RCC, TFEB can upregulate a subset of Wnt genes (Calcagnì *et al*, 2016; Di Malta *et al*, 2017; Kim *et al*, 2021). In turn, Wnt‐induced TFEB nuclear translocation may establish a positive feedback loop that sustains this oncogenic signalling cascade (Kim *et al*, 2021). However, in the liver, TFEB transcriptionally induces Sox9, inhibiting hepatocyte differentiation while promoting biliary gene expression to accelerate neoplastic transformation into cholangiocarcinoma (Pastore *et al*, 2020). This suggests that the ability of TFEB to drive tumorigenesis likely proceeds through its effects on cell division, cell fate, and differentiation, rather than on macro‐autophagy.

By contrast, it seems likely that the ability of amplified or hyperactive TFEB to promote the growth of established tumours is partly downstream of macro‐autophagy induction, which enables pro‐survival shifts in metabolism. Indeed, TFEB‐dependent upregulation of ATGs promotes metabolic reprogramming in pancreatic ductal adenocarcinoma (PDAC) to maintain a sufficient intracellular pool of amino acids (Perera *et al*, 2015). Likewise, AMPK‐mediated activation of TFEB can sustain the proliferation of pulmonary adenocarcinoma cells in the face of glucose starvation (Eichner *et al*, 2019).

Along with autophagy‐dependent metabolic reprogramming, cancer cells uniquely exhibit the concomitance of the otherwise mutually exclusive programmes of TFEB‐driven catabolism and mTOR‐driven anabolism. In fact, in tumours from patients and mouse models BHDS, RCC, PDAC, and melanoma, amplified and hyperactive TFEB transcriptionally induces the expression of RagD, thereby sustaining the proliferation of cancer cells by priming them for efficient lysosomal recruitment and re‐activation of mTORC1 upon refeeding and consequent replenishment of the intracellular amino acid pool (Di Malta *et al*, 2017). Moreover, in the renal tumours from mouse models of tuberous sclerosis complex (TSC), the absence of Tsc2 inhibits FLCN and thus promotes RagC/D^GTP^ formation, establishing a non‐canonical means of preventing mTORC1‐mediated phosphorylation of TFEB, thereby driving its nuclear translocation (Alesi *et al*, 2021). In turn, TFEB hyperactivity promotes renal cell proliferation and increases tumour volume (Asrani *et al*, 2022), likely through concurrent induction of mTOR activity and ATG transcription. Notably, however, the effect of this co‐occurrence on cancer cell survival depends on the tissue in which both mTORC1 and TFEB are hyperactive; for example, TFEB‐mTORC1 hyperactivity can allow tumour‐associated macrophages to outcompete cancer cells (Zhang *et al*, 2023).

Macro‐autophagy can also selectively deliver fragments of lipid droplets to the lysosome for lysosomal acid lipase (LAP)‐mediated degradation (lipophagy), providing another paradigm for understanding the diverse roles of TFEB in metabolic homeostasis. By transcriptionally upregulating the ATGs, TFEB can induce lipophagy and thus constitutes a crucial regulatory axis in lipid catabolism. In fact, starvation promotes nuclear translocation of the *Caenorhabditis elegans* orthologue of TFEB, hlh‐30, whereupon it stimulates transcription of the LAPs, LIPL1 and LIPL3 (O'Rourke & Ruvkun, 2013), while, in mammalian cells, starvation enhances the ability of TFEB to increase its own transcription, thereby establishing a positive feedback loop that promotes and sustains lipophagy (Settembre *et al*, 2013). In mammalian cells, TFEB‐mediated upregulation of LAPs can inhibit foam cell formation and thus disrupt atherosclerosis (You *et al*, 2020).

However, TFEB can also coordinate lipid catabolism independently of lipophagy. Disorders of metabolism thus provide a paradigm for understanding the lipophagy‐independent role of TFEB‐mediated regulation of lipid catabolism. Indeed, hepatic overexpression of TFEB in diet‐induced and genetic mouse models of obesity prevents weight gain and metabolic syndrome (Settembre *et al*, 2013). Perhaps this arises, in part, from the ability of TFEB to transcriptionally upregulate PGC‐1(alpha) and thereby promote PPAR alpha (PPARA)‐mediated upregulation of lipid catabolism, as well as the browning of adipose tissue (Evans *et al*, 2019). Moreover, the ability of TFEB to mitigate the progression of atherosclerosis is partly due to its induction of the cholesterol efflux regulatory protein, ATP‐binding cassette transporter (ABCA1), and concomitant downregulation of the class B scavenger receptor, CD36 (Li *et al*, 2016a). These non‐lysosomal impacts of TFEB on lipid metabolism may also be relevant to its roles in tumorigenesis.

### TFEB as a regulator of host cell defence against pathogens

Macro‐autophagy can selectively deliver pathogens to the lysosome for degradation (xenophagy). By transcriptionally upregulating the ATGs, TFEB can induce xenophagy and thus constitutes a crucial regulatory axis in host cell defence against pathogens. Indeed, *Staphylococcus aureus* toxins that form pores in the enterocytes of *C. elegans* stimulate the TFEB orthologue, hlh‐30, to induce the transcription of genes encoding lysozymes, anti‐microbial peptides, C‐type lectins (Visvikis *et al*, 2014), and the xenophagy programme (Chen *et al*, 2017) in a manner that is necessary for *S. aureus* clearance. In a similar vein, TFEB‐induced lysosomal biogenesis enhances pathogen clearance during both FC(gamma)‐independent and ‐dependent phagocytosis in mammalian macrophages (Pastore *et al*, 2016).

**Figure 6:**
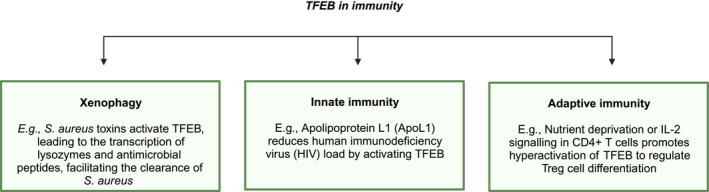
TFEB as a regulator of host cell defence against pathogenic invasion TFEB not only promotes xenophagic clearance of pathogens but also contributes to innate immunity and the development of adaptive immunocompetence. Accordingly, infectious burden can elicit post‐translational modifications that alter TFEB activity. Created with BioRender.com.

However, diversification of the metazoan lineage conferred novel functions on TFEB, which is thus also able to coordinate host innate and adaptive immunity independently of its ability to promote xenophagy (Fig 6).

#### TFEB as an autophagy‐independent regulator of host innate immunity

Infectious disease provides a paradigm for understanding the cytoprotective role of TFEB in facilitating host innate immune defence against pathogens. The outer membrane component of gram‐negative bacteria, lipopolysaccharide (LPS), can activate macrophagic AMPK to phosphorylate FNIP1, reducing lysosomal RagC/D^GDP^‐mediated recruitment of TFEB, which increasingly translocates to the nucleus, where it upregulates the transcription of genes encoding pro‐inflammatory cytokines (El‐Houjeiri *et al*, 2019). In fact, TFEB overexpression can suppress the capacity of LPS to kill macrophages through ligation of Toll‐like receptor 4 (TLR4) (Schilling *et al*, 2013). The ability of TFEB to mitigate lipotoxicity in the context of pathogenic infection indicates that its role in host innate immunity emerges, in part, from its regulation of the cross‐talk between lysosomal function, lipid catabolism, and the immune response. Indeed, TFEB depletion increases the susceptibility of enterocytes to injury and consequent colitis by downregulating apolipoprotein A1 (ApoA1) in a manner reminiscent of Crohn's disease (Murano *et al*, 2017). Furthermore, apolipoprotein L1 (ApoL1) reduces human immunodeficiency virus (HIV) load by activating TFEB (Taylor *et al*, 2014; Fig 6).

Infectious burden creates an intracellular environment that encourages the post‐translational addition of moieties to TFEB, and interaction of proteins with it, in order to modulate its activity in such a way as to relieve the causative agent. For instance, infection of macrophages with *Mycobacterium tuberculosis* or HIV stimulates IRGM and GABARAP to inhibit mTORC1‐mediated phosphorylation of TFEB, driving its nuclear translocation (Kumar *et al*, 2020). Furthermore, accumulation of cytosolic DNA, often indicative of pathogenic invasion, promotes the nuclear translocation of TFEB, and consequently, its ability to transcriptionally induce lysosomal biogenesis, which can, in turn, activate STING, TBK1, IRF3, and IRF7 to upregulate a subset of interferon‐stimulated genes (ISGs; Hasan *et al*, 2013). Perhaps this enables an early response to viral infection before a fully fledged interferon response can be initiated. In agreement with this hypothesis, (beta)‐coronavirus infection activates CaN, thereby dephosphorylating TFEB, which transcriptionally upregulates a subset of cytokines independently of the interferon response (Contreras *et al*, 2023).

By contrast, other pathogens establish an intracellular environment that facilitates the post‐translational addition of moieties to TFEB, and interaction of proteins with it, in order to suppress its activity in such a way as to promote their own replication and dissemination. Interestingly, HIV activation of TLR8 actually stimulates TFEB, eliciting a transient induction of macro‐autophagy that permits HIV replication (Campbell & Spector, 2013). Yet, as sustained macro‐autophagy can lead to xenophagy, chronic HIV infection suppresses macro‐autophagy through Nef‐mediated hyperactivation of mTORC1, which recruits and phosphorylates TFEB, such that it remains sequestered and transcriptionally inactive within the cytosol (Campbell & Spector, 2013).

#### TFEB as an autophagy‐independent regulator of host adaptive immunity

The development of immunocompetence also provides a paradigm for understanding the role of TFEB in facilitating host adaptive immunity. In the host adaptive defence against pathogens, dendritic cells initiate the immune response by presenting foreign antigens on either major histocompatibility complex (MHC) class I or II to CD4^+^ T cells, which, upon activation, express CD40 ligand (CD40L) to bind CD40 on B cells and promote their secretion of antibodies that bind such antigens. During dendritic cell maturation, TFEB acts as a molecular switch, suppressing exogenous antigen cross‐presentation while preferentially promoting MHC class II presentation (Samie & Cresswell, 2015). Furthermore, TFEB is necessary for the transcriptional upregulation of CD40L in activated CD4^+^ T cells (Huan *et al*, 2006), such that the depletion of TFEB disrupts the humoral immune response. Moreover, nutrient deprivation or IL‐2 signalling in CD4^+^ T cells promote post‐translational hyperactivation of TFEB, which orchestrates the differentiation of regulatory T (T_reg_) cells through upregulation of the genes that specify T_reg_ fate and maintain mitochondrial fitness and function, and downregulation of genes involved in cytokine secretion and lipid catabolism, without affecting macro‐autophagy (Xia *et al*, 2022).

In summary, this review considers the intricate mechanisms through which TFEB assimilates signals from the intracellular milieu to initiate tailored adaptive responses to the stresses imposed by various pathological states. We review the multifaceted, context‐dependent roles of TFEB‐mediated regulation of lysosomal biogenesis, autophagy, mitochondrial homeostasis, and immune responses in the context of various diseases. Nevertheless, substantial gaps persist in our understanding of the complex mechanisms regulating the activity of TFEB. Thus, further research is needed to elucidate its functions and regulation, which may reveal therapeutic prospects across a myriad of pathological conditions.

## Outstanding questions

Although MiT/TFE family members have been extensively studied, many questions remain not only about the understudied transcriptional and post‐transcriptional mechanisms controlling their activity but also about the post‐translational regulation and role of TFEB in health and disease (Box A).

Box A **In need of answers**

- Do the activity and function of TFEB homodimers differ from those of heterodimeric TFEB?
- What are the precise mechanisms that promote the nuclear translocation of TFEB downstream of its acetylation and PARsylation?
- Why can TSC regulate mTORC1‐dependent modulation of TFEB subcellular localisation in some cells (Alesi *et al*, 2021; Asrani *et al*, 2022), but not others (Napolitano *et al*, 2020)? Are such pathways physiological negative feedback loops that constrain stress‐induced regulation of TFEB, or simply cell‐type specific?
- What are the roles of glial TFEB in neurodegenerative disease? Why do glia often elicit post‐translational modifications that promote TFEB gain of function rather than loss of function?
- Which oncogenic consequences of TFEB hyperactivity are necessary to overcome the otherwise inhibitory effects of macro‐autophagy induction on tumorigenesis?
- How can pharmacological agents that aim to augment TFEB function in patients with LSDs or disorders of proteostasis avoid TFEB‐mediated tumorigenesis?
- How can pharmacological agents that aim to depress TFEB function in patients with cancer avoid the aberrant proteostasis, organelle biogenesis, and immune response that may result?

## Author contributions

**Michael Takla:** Conceptualization; data curation; formal analysis; writing – original draft; writing – review and editing. **Swati Keshri:** Conceptualization; data curation; formal analysis; writing – original draft; writing – review and editing. **David C Rubinsztein:** Conceptualization; formal analysis; supervision; funding acquisition; project administration; writing – review and editing.

## Disclosure and competing interests statement

DCR is a consultant for Aladdin Healthcare Technologies Ltd., Mindrank AI, Nido Biosciences, Drishti Discoveries, and PAQ Therapeutics. None of the other authors declare that they have no conflict of interest.
